# The Influence of Tissue Ischemia Time on RNA Integrity and Patient-Derived Xenografts (PDX) Engraftment Rate in a Non-Small Cell Lung Cancer (NSCLC) Biobank

**DOI:** 10.1371/journal.pone.0145100

**Published:** 2016-01-05

**Authors:** Francesco Guerrera, Fabrizio Tabbò, Luca Bessone, Francesca Maletta, Marcello Gaudiano, Elisabetta Ercole, Laura Annaratone, Maria Todaro, Monica Boita, Pier Luigi Filosso, Paolo Solidoro, Luisa Delsedime, Alberto Oliaro, Anna Sapino, Enrico Ruffini, Giorgio Inghirami

**Affiliations:** 1 Department of Thoracic Surgery, University of Torino, 10126, Torino, Italy; 2 Department of Molecular Biotechnology and Health Science and Center for Experimental Research and Medical Studies, University of Torino, 10126, Torino, Italy; 3 Department of Pathology and Laboratory Medicine, Weill Cornell Medical College, New York, NY, 10021, United States of America; 4 Department of Medical Sciences, University of Torino, 10126, Torino, Italy; 5 Department of Medical Sciences, Allergology and Immunology, University of Torino, 10126, Torino, Italy; 6 Unit of Pulmonology, San Giovanni Battista Hospital, 10126, Torino, Italy; 7 Department of Pathology and NYU Cancer Center, New York University School of Medicine, New York, NY, 10016, United States of America; Ospedale Pediatrico Bambino Gesu', ITALY

## Abstract

**Introduction:**

Bio-repositories are invaluable resources to implement translational cancer research and clinical programs. They represent one of the most powerful tools for biomolecular studies of clinically annotated cohorts, but high quality samples are required to generate reliable molecular readouts and functional studies. The objective of our study was to define the impact of cancer tissue ischemia time on RNA and DNA quality, and for the generation of Patient-Derived Xenografts (PDXs).

**Methods:**

One-hundred thirty-five lung cancer specimens were selected among our Institutional *BioBank* samples. Associations between different warm (surgical) and cold (ex-vivo) ischemia time ranges and RNA quality or PDXs engraftment rates were assessed. RNA quality was determined by RNA integrity number (RINs) values. Fresh viable tissue fragments were implanted subcutaneously in NSG mice and serially transplanted.

**Results:**

RNAs with a RIN>7 were detected in 51% of the sample (70/135), with values of RIN significantly lower (OR 0.08, P = 0.01) in samples preserved for more than 3 hours before cryopreservation. Higher quality DNA samples had a concomitant high RIN. Sixty-three primary tumors (41 adenocarcinoma) were implanted with an overall engraftment rate of 33%. Both prolonged warm (>2 hours) and ex-vivo ischemia time (>10 hours) were associated to a lower engraftment rate (OR 0.09 P = 0.01 and OR 0.04 P = 0.008, respectively).

**Conclusion:**

RNA quality and PDXs engraftment rate were adversely affected by prolonged ischemia times. Proper tissue collection and processing reduce failure rate. Overall, NSCLC BioBanking represents an innovative modality, which can be successfully executed in routine clinical settings, when stringent Standard Operating Procedures are adopted.

## Introduction

Lung cancer remains the leading cause of cancer deaths [1]. Non Small Cell Lung Cancer (NSCLC) is the most common subtype, and the clinical-pathological staging is considered the gold standard to define patients’ prognosis. Nevertheless, the correct definition of clinical outcome in each specific patient is still problematic [2]. Even though in the last decade novel therapeutic approaches, including molecular therapies, have been introduced, a comprehensive molecular-clinical characterization for each individual remains applicable only to a fraction of the patients and limited to health insurance provider policies. Lastly, even in the best settings, the results are still highly dismal.

There is an overwhelming agreement that to advance our clinical success, a detailed map of the pathogenetic mechanisms driving lung cancers is absolutely necessary. To obtain patient specific and comprehensive fingerprints the integration of multiple analytical modalities (e.g. genomic, transcriptomic, proteomic) and appropriate samples (e.g. tissue samples, plasma) [3] is required. Ultimately, the knowledge that will emerge is expected to be rapidly translated into personalized treatment protocols. Public bio-repositories of molecularly characterized and clinically annotated samples are believed to represent a highly valuable tool for the design and execution of innovative and more successful treatments.

Once bio-repository efforts are associated to Patient-Derived Xenografts, their values increase enormously. Indeed, PDX models represent the cutting edge in translational research [4] and when associated to Next-generation Sequencing (NGS) analyses provide an unmatched tool. Since the rate of PDX engraftment is linked to multiple variables, including the nature of the tumors, stage of disease and quality of tissue sampling, a rapid tissue acquisition and a correct handling of fresh specimens is absolutely critical.

Toward this end, the transfer of surgical specimens from the operating room (OR) to the surgical pathology theater requires a sharp organization, capable to implement stringent Standard Operating Procedure (S.O.P.) within an integrated multi-disciplinary network [5]. Since a timely transfer limits the ex-vivo ischemia time, considerable effort should be devoted to improve this critical step favoring the best preservation of the pathological specimen. Our purpose was to define the impact of cancer tissue ischemia time on the quality of lung cancer specimens stored in our bio-repository and how this may influence molecular high-throughput analyses and the development of new preclinical in vivo models.

Here we describe a protocol for tissue collection that envision the immediate preservation of the surgical specimen using a vacuum sealing method within the OR facility, followed by sample transport at 4°C to surgical pathology.

## Methods

### A. Patient Cohort Description

We retrospectively analyzed lung cancer specimens collected from 135 patients who underwent surgical resection from 2010 to 2012 with initial curative intent. Patients treated with preoperative protocols (*i*.*e*. chemotherapy, radiotherapy) were included. Within the biorepository matched normal lung tissue, peripheral blood mononuclear cells (PBMCs), serum and saliva samples were acquired.

### B. Informed Consent

A dedicated informed consent, concerning storage of human samples, general use in research and molecular analyses was developed. Particular emphasis has been given to the following issues:

- Purpose of the study
- Voluntary participation
- Collection of clinical and demographic data
- Privacy and confidentiality
- Benefits and risks of participation
- Re-contact for follow-up information and research results return
- Withdrawal of consent
- Secondary research project
- Sample ownership and intellectual property
- Bio-specimens and bio-fluid storage

For each patient enrolled in the Biobanking protocol, the written informed consent was collected in preoperative setting by the attending surgeon of the Thoracic Surgery Department and the patient was recorded in the BioBank Registry. This study was approved by Institutional Review Board of the Azienda Ospedaliera Universitaria, Citta’ della Salute e della Scienza di Torino.

### C. Collection Protocol

Saliva samples were collected preoperatively with Oragene•DNA (OG-500) for long-term storage. Blood samples (10 cc) were peri-operatively acquired in BD Vacutainer tubes (2 heparinized and 2 non-heparinized tubes) and processed. PBMCs, plasma and serum, were harvested and appropriately stored. The tissue bank team was alerted the day before the surgery.

The specimens were preserved and transferred to the pathology lab using the vacuum packing and cooling (VPAC) procedure [6, 7]. Briefly, in the OR, explanted surgical specimens were immediately placed into beta-ray sterilized plastic bags and vacuum-sealed using the TissueSAFE machine (Mod. VAC 10, by Milestone, Bergamo, Italy; www.milestonemedsrl.com), as standard procedure in our Institution [8]. Specimens were then preserved and brought to the pathology laboratories, in chilled (at 4°C) plastic box, and further stored at 4°C prior processing.

Routine histo-pathological procedures were integrated with BioBanking protocol (S1 Fig). Each specimen was oriented, measured, and described, being aware that the collection would not affect the pathological diagnosis. Then, tumor masses were excised and sampled. At the same time, normal lung tissue was taken from a distal uninvolved area (S2 Fig). Tissue fragments were stored in different media: in OCT (Sukura Finetek, Torrance, CA) for frozen tissue cutting, RNA*later*^®^ Stabilization Solution (Life Technologies) for RNA extraction and snap-frozen and then kept in -80° refrigerator. Lastly, small tissue fragments (2X2X2 mm) were placed in a freezing medium (59% RPMI, 30% FBS, 10% DMSO, 1% PEN-STREP) and then stored in liquid nitrogen. Representative diagnostic samples underwent routine histopathological processing procedures. Related patient informations (storage and cryopreservation times and clinical, demographics and histopathological data) were stored within the BioBank Registry.

### D. DNA/RNA Extraction And Quality Assessment

Genomic DNA was extracted with standard phenol-chloroform extraction method [9].

Tissue samples were homogenized in trizol and RNA was extracted as for manufacturer’s instructions. To eliminate any genomic DNA contamination, when present, RNA samples were subjected to deoxyribonuclease treatment and re-extracted using phenol-chloroform method [10].

The total DNA and RNA were quantified with NanoDrop 2000c (Thermoscientific) instrument and the absorbance ratios at 260nm/280nm and 260nm/230nm were recorded. Total RNAs (1 μL) were analyzed in an Agilent 2100 Bioanalyzer (Agilent Technologies, Inc., Santa Clara, CA) using RNA Nano LabChips (Caliper Technologies Corporation, Hopkinton, MA). RNA integrity number (RIN) values were established as empirical measurements of RNA integrity.

### E. cDNA Synthesis And qRT-PCR

Total RNA was reverse-transcribed before real-time PCR amplification. 1 ng of total RNA was treated with DNAseI recombinant, RNase-free (Roche Diagnostics, Mannheim, Germany) and reverse-transcribed using the Superscript First-Strand Synthesis System for RT-PCR kit (Invitrogen Life Technologies) according to the manufacturer’s instructions. RT-qPCR was performed with a Thermal iCycler (Bio-Rad) using the iQ SYBR Green Supermix (Bio-Rad) according to the manufacturer’s instructions. The PCR cycling conditions were as follows: 95°C for 5 minutes, followed by 40 cycles at 94°C for 10 seconds then 60°C or 62°C for 30 seconds. To confirm the amplification specificity, PCR products were subjected to analysis of melting curve, linearity, and slope of standard curve using CFX software (Bio-Rad). All PCR assays were performed in triplicate.

mRNA expression levels were evaluated for 3 different housekeeping genes: GAPDH, ACTIN and HUPO. Primer sets were designed using Primer3 (S1 Table).

### F. Multiplex PCR

DNA integrity was estimated by a multiplex PCR with a set of 5 pairs of oligoprimers designed to amplify different DNA targets (100, 200, 300, 400 and 600 bp) (S2 Table) [11]. Primers were added in 1:1:1:2 ratio to obtain PCR products with equal intensity after agarose gel separation. DNA amplification was performed as following: preactivation 7 min at 95°C (1 cycle) followed by 40 cycles: denaturation 45 sec at 95°C, annealing 50 sec at 60°C, extension 1.30 min at 72°C with final extension cycle of 15 min at 72°C. PCR products were separated by agarose gel electrophoresis (2% agarose in TBE buffer 1%).

### G. Patient Derived Xenografts (PDX)

NOD.Cg-Prkdcscid Il2rgtm1Wjl/SzJ (NSG) mice were kindly provided by L. Shultz from The Jackson Laboratory and bred within the Molecular Biotechnology Center (MBC) Animal Resource, under strict specific and opportunistic pathogen free (SOPF) conditions.

The animal protocol for this study was reviewed and approved by the Animal Committee of the University of Torino.

Briefly PDXs were established using fresh or frozen pathological tissue fragments, only when sufficient material was available for routine diagnostic and molecular analyses. Tumor-graft samples were cut into multiple 2x2x2 mm pieces (multiple pieces/specimen) in complete media. Six- to eight-week-old NSG were first anesthetized (Rompun 0.05μl/g e Zoletil 1.6μg/g i.m.), and their dorsal region was sterilized (70% ethanol). A skin incision (0.3 cm) was subsequently made along the dorsal midline retronuchal region and a small pocket was created by blunt dissection. Multiple tumor-graft tissue fragments (2–4) were transferred into each subcutaneous pocket using blunt-ended forceps. The cut edges were sealed with a single metal clip. Mice were regularly checked until they became vigil. Implanted animals were housed in same-sex groups. Implant growth was assessed by palpation and when required tumor masses were harvested (<1.5 cm^3^). Recipient animals were checked regularly and sacrificed at early sign of distress. At harvesting, mice were euthanized in a CO2 chamber and tumorgrafts were collected for histologic evaluation, molecular studies, re-grafting, or snap-frozen in liquid nitrogen.

### H. Study Outcomes And Statistical Analysis

Categorical data are presented as number (percentage, %), continuous data are presented by their median (interquartile range, iqr).

The RIN was calculated in all specimens as surrogate of tissue preservation. A RIN of ≥7 was set as a cutoff to perform RNA microarray expression arrays and RNAseq analyses.

PDX engraftment was considered successful when tumor masses were generated after at least two passages and their pathological features were confirmed by histology and immunohistochemistry. Pearson chi-square test and Fisher’s exact test, when appropriate, were used to evaluate the differences in the engrafted and non-engrafted groups: tumor dimension, histology, tumor grading, pathological TNM stage, resection status, tumor-infiltrating lymphocytes (TIL), presence of microvascular invasion and surgical duration were determined.

The influence of tissue ischemia times on RIN quality and PDX engraftment success were the primary end-points of the study. The *warm ischemia* time was intended as the total surgical duration and the *ex-vivo (cold) ischemia time* was defined as the time frame between the acquisition of specimens in the surgical theatre and the time of their cryopreservation.

As a second endpoint, we evaluated RNA expression levels and genomic DNA integrity in a randomly chosen subset of specimens (*sample* command in STATA): we selected 3 samples with low quality RIN and 3 with high quality RIN among each ischemia time classes. Gene-expression results were evaluated as absolute expression in terms of Quantification cycles (Cq mean). Differences between the groups were investigated by Wilcoxon-Mann-Whitney test. The year of procurement was also analyzed.

For the purpose of our research, we grouped cases in four classes according to ex-vivo ischemia times: ≤1 hours, 1–2 hours, 3–5 hours, 6–9 hours and ≥10 hours.

The association between RNA quality (RIN of ≥7) or PDX engraftment rates and individuated groups was assessed using logistic regression model. To avoid possible confounding influences, an adjusted multivariate logistic regression model including the following surgical and pathological variables was performed: histological subtypes (adenocarcinoma as reference), tumor grading (G1 as reference), pathological TNM stage (Stage I as reference) and surgery duration (hours, as continuous).

Odds ratio (OR) and the corresponding 95% confidence intervals (95% CIs) were provided for each model. All statistical analyses were assessed using STATA (version 12.1).

## Results

### A. Clinical And Pathological Features

A total of 135 lung cancer specimens were investigated. Table 1 summarizes their clinical, surgical and pathological characteristics. Median age at surgery was 69 years (IQR 64–75), patients were more frequently male (92, 68%) and smokers (106, 81%). Median surgical duration (IQR 1–3) and median ex-vivo ischemia time (IQR 1–6) were both of 2 hours. Median tumor size was 3 cm (IQR 2–5) and tumor pathological TNM stages were as follow: stage IA 32 (23%), IB 13 (10%), IIA 21 (15%), IIB 13 (10%), IIIA 37 (27%) IIIB 1 (1%), IV 18 (13%). Adenocarcinoma was the most common histological subtype (89, 66%) followed by squamous cell carcinoma (31, 23%), large cell carcinoma (7, 5%), carcinoid (4, 3%), sarcomatoid (2, 2%), adenosquamous (1, 1%) and small-cell (1, 1%). According to Travis adenocarcinoma classification [12], we observed 44 (55%) acinar adenocarcinoma, 21 (26%) solid, 10 papillary, 3 mucinous, 2 lepidic and 9 NOS. Ninety-five (70%) patients were submitted to lobectomy, 14 (10%) to bilobectomy, 13 (10%) to pneumonectomy and 13 to sub-lobar resection; positive resection margins were observed in 13 cases (10%, 10 R1 and 3 R2).

**Table 1:** Pathological characteristics of interest for 135 NSCLC samples.

	Entire cohort (n = 135)		PDX cohort (n = 63)	
	n	%	n	%
**Gender (male)**	92	68	43	68
**Age (years) (median–iqr**^§^**)**	69	64–75	68	63–74
**Smoke (yes)**	106	81	52	85
**Type of intervention and surgical approach**				
Bilobectomy or Pneumonectomy	27	20	12	19
Lobectomy	95	70	47	75
Sublobar resection	13	10	4	6
**RIN>7**	70	51	29	46
**Surgical Time (h) (median–iqr)**	2	1–3	2	1–3
**Surgical Time >2 h (yes)**	64	47	28	44
**Ex-vivo ischemia time (h) (median–iqr)**	2	1–6	2	1–3
**Ex-vivo ischemia time >2 h (yes)**	49	36	12	19
**Tumor size (cm) (median–iqr)**	3	2–5	3	2–5
**Histology**				
Adenocarcinoma	89	66	41	65
Carcinoid	4	3	-	-
Combined	1	1	-	-
Large cell carcinoma	7	5	3	5
SCLC	1	1	-	-
Sarcomatoid	2	1	1	2
Squamous	31	23	18	29
**Adenocarcinoma subtypes**				
Acinar	44	55	19	50
Lepidic	2	3	1	3
Mucinous	3	4	1	3
Papillary	10	13	4	11
Solid	21	26	13	34
**Histology grading**				
G1	3	2	-	-
G2	70	54	36	57
G3	56	43	27	43
**Pathologic TNM stage**				
I	45	33	17	27
II	34	25	18	29
III	38	28	16	25
IV	18	13	12	19
**Resection status (R1)**	13	10	5	8
**Vascular invasion (yes)**	95	77	44	72

^§^ interquartile range

### B. RIN Quality

**Fig 1:**
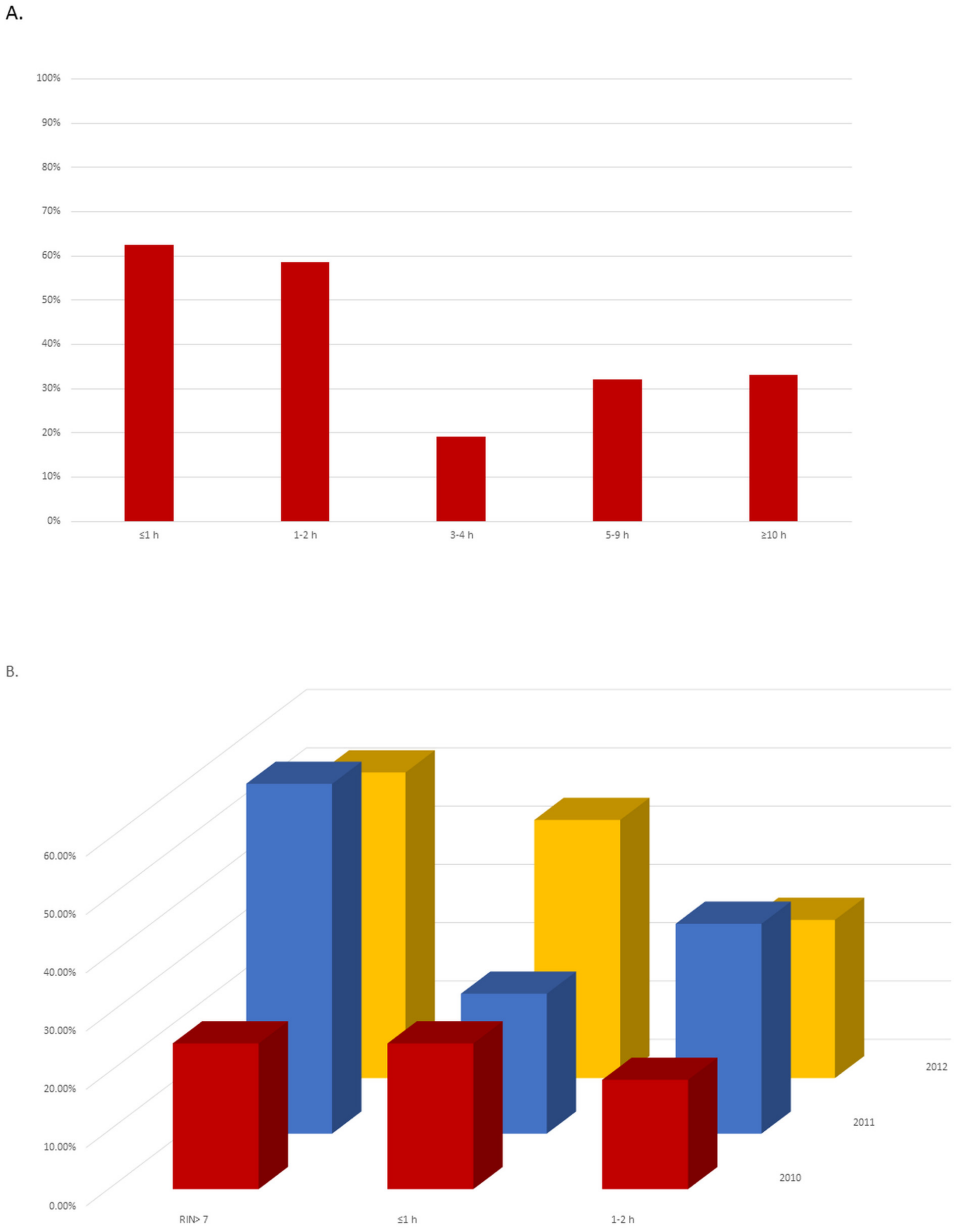
A- Percentage of samples suitable for gene expression arrays (RNA integrity number [RIN] of ≥7) at different ex-vivo procurement times (time from surgical removal to cryopreservation) for ≤1 hours, 1–2 hours, 3–4 hours, 5–9 hours and ≥10 hours. B- Relation between ex-vivo procurement times (<1 and 1–2 hours, from excision to cryopreservation) and suitability for gene expression arrays (RNA integrity number [RIN] of ≥7) and time periods 2010, 2011, and 2012.

**Fig 2:**
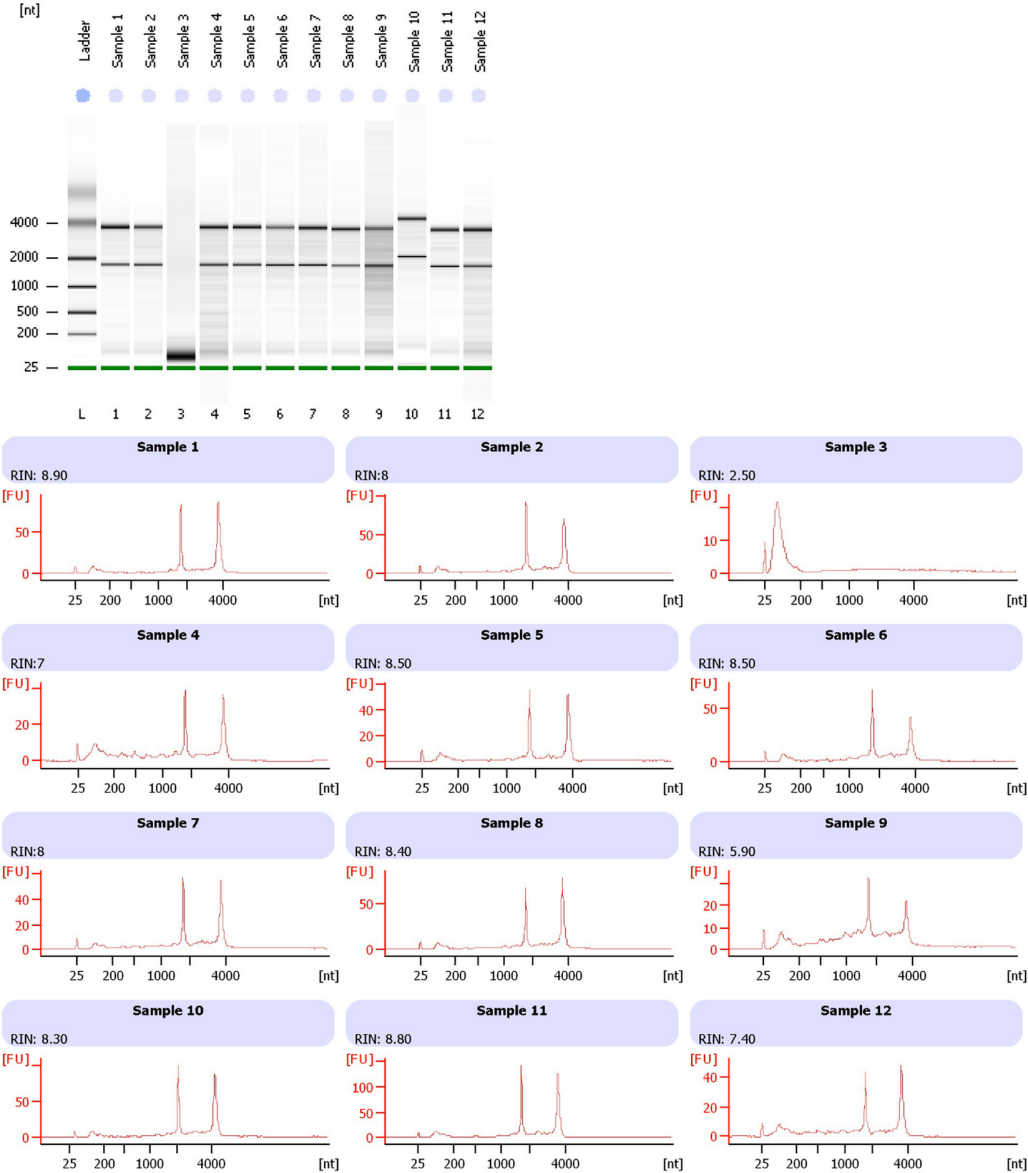
Integrity of total RNA isolated from banked fresh-frozen lung cancer samples. Representative electropherograms (Agilent 2100 Bioanalyzer) show RNA integrity measured by RNA integrity number (RIN) of 12 representative samples (bottom).

A RIN ≥7 was observed in 51% of the sample (70/135): the percentage of samples with a high RNA quality according to the different ex-vivo ischemia time is illustrated in Fig 1A. Representative electropherograms show the RNA integrity measures of 12 representative samples (Fig 2, bottom panel) obtained from banked fresh-frozen lung cancer samples.

Using a multivariate adjusted model we showed that samples stored after different intervals display a decline of RIN value soon after 2 hours of ex-vivo ischemia time (cold ischemia): 3–4 hours (OR 0.08, P = 0.044), 5–9 hours (OR 0.15, P = 0.011) and ≥10 hours (OR 0.25, P = 0.022) (Table 2). Contrariwise, surgical time (warm ischemia) did not influence RNA quality.

**Table 2:** Correlation of RNA quality (RIN>7) and ex-vivo ischemia time, surgical time and pathological variables. Multivariate logistic regression analysis on 135 NSCLC samples.

	Odds Ratio	Std. Err.	P	[95% Conf.Interval]	
				lower	upper
**ex-vivo ischemia time (cold ischemia) (≤1 hours as reference)**					
*1–2 h*	0.61	0.32	0.343	0.22	1.69
*3–4 h*	0.08	0.10	0.044	0.01	0.94
*5–9 h*	0.15	0.11	0.011	0.03	0.65
*≥10 h*	0.25	0.15	0.022	0.08	0.82
**Surgical duration (warm ischemia) (hours, <2 as reference)**					
*>2h*	2.35	1.29	0.120	0.80	6.87
**Histology (adenocarcinoma as reference)**					
*Squamous*	1.39	0.72	0.519	0.51	3.82
*Other*	0.18	0.16	0.056	0.03	1.05
**Tumor Grading (G1 as reference)**					
*G2*	1.35	2.01	0.841	0.07	25.12
*G3*	2.34	3.49	0.569	0.13	43.70
**pTNM Stage (Stage I as reference)**					
*II*	0.25	0.14	0.016	0.08	0.77
*III*	0.33	0.18	0.046	0.11	0.98
*IV*	0.30	0.19	0.064	0.08	1.07

No difference in the proportion of samples with high-quality RNA was demonstrated according to the year of storage, histology and tumor grade: Fig 1B shows the percentage of tumor specimens with RIN of ≥7 and the ex-vivo procurement time, according to biobanking procurements. Inversely, a high pTNM stage was associated with low RIN values (<7, P< 0.05).

### C. mRNA Expression Level

**Fig 3:**
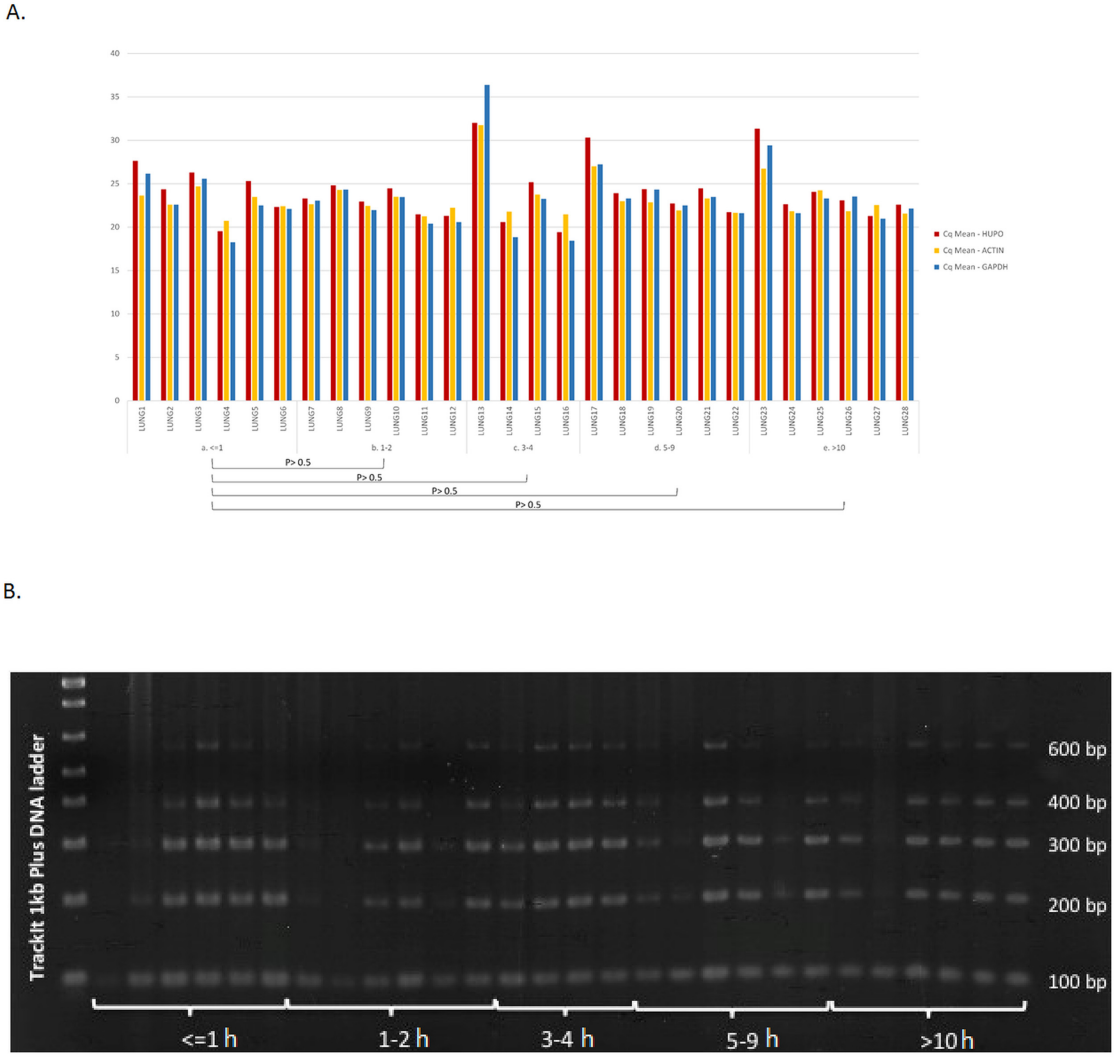
A- Cq expression levels of housekeeping genes in randomly selected 28 samples representative of all the time categories. B- Gel electrophoresis of 28 genomic DNA amplified with multiplex PCR amplifying at different numbers of base paires (100 bp, 200 bp, 300 bp, 400 bp, 600 bp).

Twenty-eight cases were randomly selected to assess the mRNA quality by evaluating expression levels of housekeeping genes. As shown in Fig 3A, the Cq values obtained are between 18 and 37. Cq with more than 25 cycles have been observed in samples with RIN <4, whereas in samples with RIN values within 4–10, these genes could efficiently detected with less cycles (20 to 25 cycles). Samples with lower expressions were preferentially observed in the group with ≥ 3 hours of ex-vivo ischemia time.

### D. High-Molecular-Weight (HMW) Genomic DNA Integrity

The same 28 samples used for RNA determination were also evaluated for their genomic DNA integrity (Fig 3B). The presence of bands at each size was used as a surrogate of quality of the DNA, considering the amplification through 600bp as the highest quality DNA value. Samples with lower RIN for each timeframe groups had also poor DNA quality. Observed DNA quality seems to be similar in all ranges of ex-vivo ischemia time.

### E. PDX Engraftment

Sixty-three primary tumors (41 adenocarcinoma, 18 squamous cell carcinoma, 3 large cell carcinoma and 1 sarcomatoid carcinoma) were implanted, with an overall total engraftment rate of 33% (21/63).

Tumors with larger dimensions (>3 cm; 23/25 vs 16/38, P< 0.001) and a higher pathological stage (III-IV; 15/25 vs 13/38, P = 0.048) were more frequently implanted. Squamous cell carcinoma (12/25 vs 6/38, P = 0.017) and tumor with resection margins involvement (R1-R2; 4/25 vs. 1/38, P = 0.064) also more frequently engrafted. Among adenocarcinoma, solid histology was associated with successful engraftment (6/11 vs. 7/27, P = 0.09). Conversely, high tumor grading (G3; 12/25 vs. 15/38), positive TIL (16/17 vs. 30/34) and presence of microvascular invasion (4/23 vs. 13/38) had similar frequency in the engrafted and non-engrafted groups.

Multivariate adjusted model showed the correlation between both prolonged ex-vivo ischemia (cold ischemia longer than 10 hours), extended surgical time (warm ischemia longer than 2 hours) with a lower engraftment rate (P = 0.047 and P = 0.008 respectively, Table 3).

**Table 3:** Correlation of PDX engraftment and ex-vivo ischemia time, surgical time and pathological variables. Multivariate logistic regression analysis on 63 NSCLC cases.

	Odds Ratio	Std. Err.	P	[95% Conf. Interval]	
				lower	upper
**ex-vivo ischemia time (cold ischemia) (<10 hours as reference)**					
*≥10 h*	0.09	0.11	0.047	0.01	0.97
**Surgical duration (warm ischemia) (hours, <2 as reference)**					
*>2 h*	0.04	0.05	0.008	0.01	0.42
**Histology (adenocarcinoma as reference)**					
*Squamous*	8.55	7.14	0.01	1.67	43.88
*Other*	0.89	1.18	0.931	0.07	12.06
**Tumor Grading (G2 as reference)**					
*G3*	1.39	0.95	0.629	0.36	5.30
**pTNM Stage (Stage I as reference)**					
*II*	2.90	2.91	0.288	0.41	20.67
*III*	2.52	2.79	0.404	0.29	22.08
*IV*	8.06	8.68	0.053	0.97	66.58

Contrariwise, squamous cell histology (OR 8.55; P = M 0.01) and high tumor stage were associated with the engraftment success (OR 8.06; P = 0.053).

We further investigated frozen tumor samples PDX engraftment rate. Of the 21 PDX lines generated, we chose 8 tumors and we implanted the corresponding primary frozen samples harvested at the surgery time. Four of the eight samples (50%) efficiently grew, generating a PDX line comparable with the fresh-derived one.

## Discussion

The objective of our study was to evaluate the overall tissue quality of lung cancer specimens stored in our bio-repository. We also aimed to delineate the influence of the ischemia time on lung cancer specimens and how ischemia could influence molecular high-throughput analyses and the realization of preclinical in vivo models.

The results of our study suggest that (1) the RIN value is significantly influenced by ex-vivo ischemia time, tumor grading and pTNM stage; (2) Patient-Derived Xenografts engraftment-rate success is also associated with low ex-vivo ischemia-time, low surgical duration (warm ischemia time) and squamous-cell histology; (3) mRNA housekeeping genes expression levels and high-molecular-weight (HMW) genomic DNA integrity are related with RIN score and can be used as reproducible biomarkers for tissue preservation.

By implementing Standard Operating Procedures (SOPs) we were able to cryopreserve 135 primary lung cancer specimens with the goal of generating a large high-quality biorepository, and without compromising histopathological diagnosis. Each step in this process is critical: this shed light on the role that is played by an organized team and a correct triage evaluation of the sample in the Pathology Room. The VPAC system, already proven to preserve specimens integrity, was adopted as gold standard procedure in order to obtain viable tissues [6–8]. However, while in breast cancer tissues RNA, DNA and proteins may be optimally preserved for >24h [7, 13], we here showed that lung tissues are much more sensible to this time. This may suggest that ex-vivo ischemia may influence cell viability and molecular results depending on the origin of tissues.

There is a general acceptance on the importance of using intact RNA in gene expression analysis and RIN of >7 is considered acceptable as cut-off value for samples suitability [14,15]. In our repository, 51% of samples demonstrated RIN>7, with a favorable trend over the years. This result lies in between those reported for prostate cancer (60–81% [16]), colon cancer (80% [17]) and those described in pancreatic cancer (40% [14]). Possible explanations for these differences could be due to the heterogeneity of cellular content, dissimilar degradation of different tissues, inter-operator variability, etc. Although several factors could affect augmented degradation of RNA, the assumption that ex-vivo ischemia-time is strictly connected with degree of RNA degradation appears to be reasonable. Here, a decline in RNA quality was observed from 3 hours after surgical resection. This result is in line with a previous report on course degradation in lung tissue: the authors found decreased nucleic acid stability from 5 hours after surgery [18]. Similarly, limited ex-vivo ischemia time seemed to correlate with optimal RNA integrity in colon cancer (6–16 hours) [17], breast cancer (3 hours) [19], prostate cancer (2 hours) [16, 20] and in pancreas cancer tissue. Long surgical time did not affect the RNA integrity, as would be expected if the tissue ischemia was the only explanation of RIN decrease. Moreover, mRNA expression levels and HMW genomic DNA integrity seemed to be partially associated with RIN score, but showed minor quality reduction when correlated with ex-vivo ischemia time. This may rescue a large fraction of the 49% of samples with lower RIN (<7%) since they can be used for classical molecular techniques. In our study, high pTNM stages were also associated with a reduced RIN score. A possible explanation could be the surgical manipulation and the subsequent RNase release as postulated form Bertilsson and Harveer regarding prostate cancer [20].

Patient-derived xenopatients are considered, nowadays, highly reliable pre-clinical mouse models. The establishment of this tool requires sophisticated research facilities and introduces technical and logistic challenges, and has been recently evaluated as a fundamental source of biological material and therapeutically relevant informations [21, 22]. The usage of fresh and frozen primary tumor samples derived from a *Biobank* resource demonstrated the feasibility of this approach, particularly when different efforts are put together in a well-integrated network. Our group takes advantage of direct implantation in mice, subcutaneously, of fresh or frozen tissue fragments derived from surgically resected patients. The engraftment rate of 33%, even substantially comparable with ones already attested (25%-35%) by other groups in a subcutaneous setting [23–25], resulted slightly lower than expected considering the major propensity of NSG model to engraftment. However we observed a quite high percentage (29%) of engrafted adenocarcinomas that usually result less prone to implantation compared to squamous cell tumors. Ischemia time influence on engraftment rate demonstrated that a prolonged surgical (warm) ischemia period is negatively associated with success of engraftment [25] rather than total (warm and cold) time. Other characteristics influence the engraftment rate: tumor dimension (>3 cm), pathological stage (III-IV) and squamous cell histology have an impact on engraftment rate, suggesting that also intrinsic biological properties of the tumor play roles in determining the PDX successful generation. The same holds true for PDX generation from frozen tumor specimens. The capability of re-generate 50% of PDX line from cryopreserved materials suggests that different elements can influence the tissue biology; however highlight the possibility to go back retrospectively in the tissue biobank and pick specific molecular-defined tumors in order to generate appropriate PDX line useful for *ad hoc* preclinical screenings.

To date, no studies have been conducted comparing different tissue preserving methods. However, considering its ability to preserve cellular morphology, epitope integrity and nucleic acids stability, we used the VPAC system for tissue storage, speculating a positive influence also on PDX engraftment rate.

Taken together these data indicates that the generation of a lung cancer biorepository is a feasible approach and requires complex network efforts integrating different bio-medical competences. Thus, well designed protocols are fundamental for proper and efficient *Biobanks*. Pathologists, oncologists, thoracic surgeons, nurses, biologists, technicians, epidemiologists and medical statisticians must collaborate in a common operative workflow to ensure not only high quality specimens, but also appropriate processing protocol, correct storage, data collection, epidemiological analyses in efficiently coordinated projects.

Our approach provides the biorepository of a precious collection of samples for wide-ranging investigation on NSCLC. In particular, high quality RNA and DNA can be used for Next-Generation Sequencing techniques, such as RNAseq, Whole Exome Sequencing and Whole Genome Sequencing, but also nucleic acids with lower quality can be rescued for standard gene expression techniques (i.e. quantitative reverse transcriptase polymerase chain reactions). Finally, the availability of fresh and frozen tumor samples represent an invaluable resource in order to generate Patient-Derived Xenografts (PDX) lines [26].

## Conclusion

The molecular stratification of NSCLC in the recent era of “precision medicine” has highlighted the importance of maintaining series of high quality specimens integrated with clinical-pathological data. These repositories can be investigated by Next-Generation Sequencing and are mandatory for the generation of stable pre-clinical models.

Here we report a reproducible and reliable approach to generate a tissue bank for NSCLC, and we provide evidence on the critical value of specimens’ quality for subsequent studies. Indeed, RNA quality and PDX engraftment rate were adversely affected by prolonged ischemia time, and thus, a correct, monitored and well-orchestrated tissue collection might reduce failure rate.

Overall, NSCLC BioBanking represents an innovative modality that can be successfully executed in a routine clinical setting within all the institutions but requires validated Standard Operating Procedures. Our work demonstrates that, implementing these procedures, the generation of a NSCLC BioBank is not only feasible but it may provide high quality tumor specimens that result to be mandatory in the “Precision Medicine” era.

## Supporting Information

S1 Fig Biobank flow-chart of Standard Operating Procedures. (TIF)

S2 Fig Lung cancer and lung normal tissue collection and preservation. (TIF)

S1 Table Primer sequences (forward and reverse) used for RT-qPCR. (DOCX)

S2 Table Primer sequences (forward and reverse) used for Multiplex PCR. (DOCX)
