# Supplementary figures and images for: The Influence of Tissue Ischemia Time on RNA Integrity and Patient-Derived Xenografts (PDX) Engraftment Rate in a Non-Small Cell Lung Cancer (NSCLC) Biobank
Source: PLoS One. 2016 Jan 5;11(1):e0145100. doi: 10.1371/journal.pone.0145100 (PMC4701130; doi:10.1371/journal.pone.0145100)

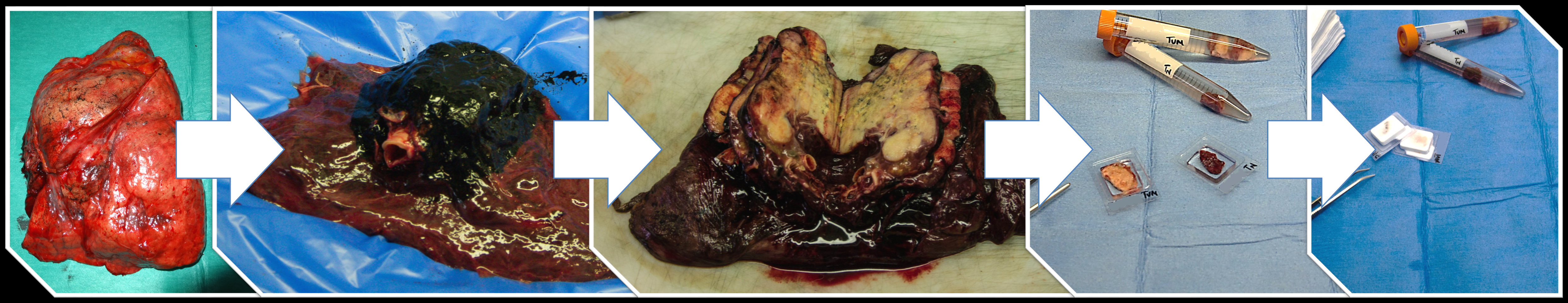

Supplement: S1 Fig — (TIF) [file pone.0145100.s001.tif]

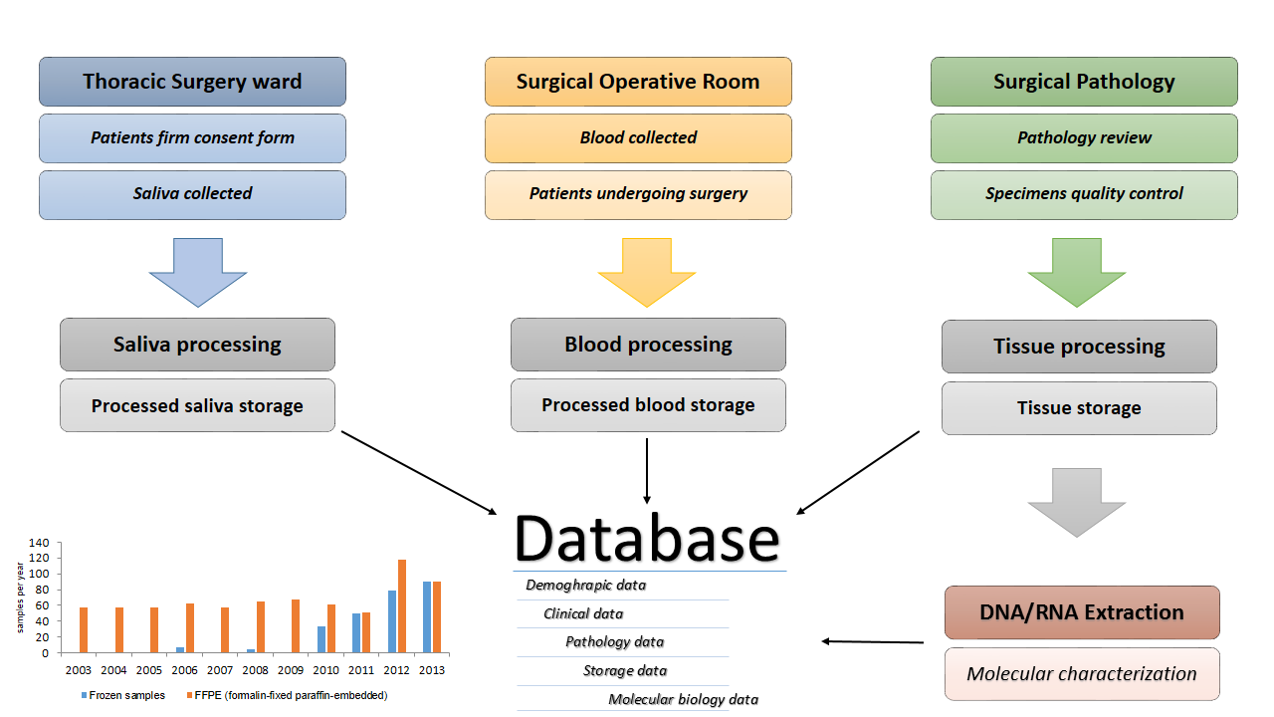

Supplement: S2 Fig — (TIF) [file pone.0145100.s002.tif]
