# Supplementary material for: The Influence of Tissue Ischemia Time on RNA Integrity and Patient-Derived Xenografts (PDX) Engraftment Rate in a Non-Small Cell Lung Cancer (NSCLC) Biobank
Source: PLoS One. 2016 Jan 5;11(1):e0145100. doi: 10.1371/journal.pone.0145100 (PMC4701130; doi:10.1371/journal.pone.0145100)
Supplement: S1 Table — (DOCX) [file pone.0145100.s003.docx]

S1 Table. Primer sequences (forward and reverse) used for RT-qPCR

| **Gene** | **Primer name** | **Primer sequence** |
| --- | --- | --- |
| GAPDH | GAPDH FW | 5'-CTCTCTGCTCCTCCTGTTCGAC-3' |
|  | GAPDH REV | 5'-TGAGCGATGTGGCTCGGCT-3' |
| ACTIN | ACTIN FW | 5'-CAGAGCCTCGCCTTTGC-3' |
|  | ACTIN REV | 5'-TCATCATCCATGGTGAGCTG-3' |
| HUPO | HUPO FW | 5'-GCTTCCTGGAGGGTGTCC-3' |
|  | HUPO REV | 5'-GGACTCGTTTGTACCCGTTG-3' |
