# Supplementary material for: The Influence of Tissue Ischemia Time on RNA Integrity and Patient-Derived Xenografts (PDX) Engraftment Rate in a Non-Small Cell Lung Cancer (NSCLC) Biobank
Source: PLoS One. 2016 Jan 5;11(1):e0145100. doi: 10.1371/journal.pone.0145100 (PMC4701130; doi:10.1371/journal.pone.0145100)
Supplement: S2 Table — (DOCX) [file pone.0145100.s004.docx]

S2 Table. Primer sequences (forward and reverse) used for Multiplex PCR

| **Gene** | **Primer name** | **bp** | **Primer sequence** |
| --- | --- | --- | --- |
| TBXAS1 | TBXAS1 ex9 FW | 100 | 5’-GCC CGA CAT TCT GCA AGT CC-3’ |
|  | TBXAS1 ex9 REV |  | 5’-GGT GTT GCC GGG AAG GGT T-3’ |
| RAG1 | RAG1 ex2 FW | 200 | 5'-TGT TGA CTC GAT CCA CCC CA-3' |
|  | RAG1 ex2 REV |  | 5'-TCATCATCCATGGTGAGCTG-3' |
| PLZF | PLZF ex1 FW | 300 | 5'-TGC GAT GTG GTC ATC ATG GTG-3' |
|  | PLZF ex1 REV |  | 5'-CGT GTC ATT GTC GTC TGA GGC-3' |
| AF4 | AF4 ex11 FW | 400 | 5'-CCG CAG CAA GCA ACG AAC C-3' |
|  | AF4 ex11 REV |  | 5'-GCT TTC CTC TGG CGG CTC C-3' |
| AF4 | AF4 ex3 FW | 600 | 5'-GGA GCA GCA TTC CAT CCA GC-3' |
|  | AF4 ex3 REV |  | 5’- CAT CCA TGG GCC GGA CAT AA-3’ |
